# Beta cell function and ongoing autoimmunity in long-standing, childhood onset type 1 diabetes

**DOI:** 10.1007/s00125-016-4087-0

**Published:** 2016-09-03

**Authors:** Georgina M. Williams, Anna E. Long, Isabel V. Wilson, Rachel J. Aitken, Rebecca C. Wyatt, Timothy J. McDonald, F. Susan Wong, Andrew T. Hattersley, Alistair J. K. Williams, Polly J. Bingley, Kathleen M. Gillespie

**Affiliations:** 1 Diabetes and Metabolism, School of Clinical Sciences, Southmead Hospital, Level 2 Learning and Research Building, Bristol, BS10 5NB UK; 2 National Institute for Health Research (NIHR) Biomedical Research Unit in Nutrition, Diet, and Lifestyle, University Hospitals Bristol National Health Service (NHS) Foundation Trust and University of Bristol, Bristol, UK; 3 Institute of Biomedical and Clinical Science, University of Exeter Medical School, Exeter, UK; 4 Institute of Molecular and Experimental Medicine, Cardiff University School of Medicine, Cardiff, UK

**Keywords:** Autoantibodies, C-peptide, Type 1 diabetes

## Abstract

**Aims/hypothesis:**

This study aimed to determine the frequency of residual beta cell function in individuals with long-standing type 1 diabetes who were recruited at diagnosis, and relate this to baseline and current islet autoantibody profile.

**Methods:**

Two hour post-meal urine C-peptide:creatinine ratio (UCPCR) and islet autoantibodies were measured in samples collected from 144 participants (median age at diagnosis: 11.7 years; 47% male), a median of 23 years (range 12–29 years) after diagnosis. UCPCR thresholds equivalent to mixed meal-stimulated serum C-peptide >0.001 nmol/l, ≥0.03 nmol/l and ≥0.2 nmol/l were used to define ‘detectable’, ‘minimal’ and ‘residual/preserved’) endogenous insulin secretion, respectively. Autoantibodies against GAD (GADA), islet antigen-2 (IA-2A), zinc transporter 8 (ZnT8A) and insulin (IAA) were measured by radioimmunoassay.

**Results:**

Endogenous C-peptide secretion was detectable in 51 participants (35.4%), including residual secretion in seven individuals (4.9%) and minimal secretion in 14 individuals (9.7%). In the 132 samples collected more than 10 years after diagnosis, 86 participants (65.2%) had at least one islet autoantibody: 42 (31.8%) were positive for GADA, 69 (52.3%) for IA-2A and 14 of 104 tested were positive for ZnT8A (13.5%). The level of UCPCR was related to age at diagnosis (*p* = 0.002) and was independent of diabetes duration, and baseline or current islet autoantibody status.

**Conclusions/interpretation:**

There is evidence of ongoing autoimmunity in the majority of individuals with longstanding diabetes. Endogenous insulin secretion continues for many years after diagnosis in individuals diagnosed with autoimmune-mediated type 1 diabetes above age 5. These findings suggest that some beta cells are protected from continued autoimmune attack in longstanding type 1 diabetes.

## Introduction

In type 1 diabetes, insulin producing beta cells are targeted for autoimmune destruction. All beta cells were formerly assumed to be destroyed but increasing evidence now indicates that endogenous insulin secretion occurs many years after diagnosis in some individuals with type 1 diabetes [1–4]. This phenomenon is associated with fewer complications, notably reduced incidence of severe hypoglycaemia [5]. These previous studies have been conducted on longstanding patients, but islet autoantibodies were not tested close to diagnosis.

The aim of this study was to determine the frequency of residual C-peptide secretion in a cohort of patients with longstanding type 1 diabetes in whom islet autoantibody measurements were carried out within 2 years of diagnosis, to establish whether persistent beta cell function and baseline or current islet autoantibody status are associated.

## Methods

### Study population

Participants were identified from the Bart’s Oxford (BOX) study. This is a prospective, population-based study that has recruited families of children who developed type 1 diabetes before the age of 21 since 1985, in the former Oxford Health Authority Region [6]. For this sub-study, further inclusion criteria were diagnosis between 1985 and 2003 with an islet autoantibody positive serum sample collected within 2 years of diagnosis.

Ethical approval was obtained from the National Research Ethics Service (NRES) East Midlands Leicester Research Ethics Committee (March 2012). Informed consent was obtained from all participants and research was carried out in accordance with the Declaration of Helsinki, as revised in 2008.

### Urinary C-peptide

Participants were asked to provide a urine sample, collected in boric acid preservative, 2 h after their main meal of the day and to return the sample on the same day by post to the University of Bristol (Bristol, UK) [7]. Urine samples were aliquoted on arrival and stored at −70°C until shipped frozen by courier to the Biochemistry Department, Royal Devon and Exeter Hospital (Exeter, UK) where the urine C-peptide:creatinine ratio (UCPCR) analysis was carried out.

Urine C-peptide was measured by electrochemiluminescence immunoassay as previously described [7]. The thresholds for UCPCR were defined as detectable (>0.001 nmol/mmol), minimal (≥0.03 nmol/mmol) and residual/preserved (≥0.2 nmol/mmol) [8].

### Islet autoantibodies

To determine current islet autoantibody status, participants collected capillary blood at home using a standard collection system (Microvette 200, Sarstedt, Nümbrecht, Germany) and returned the whole blood samples by standard post, for serum separation at the University of Bristol. These samples, along with serum collected from the same individuals within 2 years of diagnosis were tested for islet autoantibodies to GAD (GADA), islet antigen-2 (IA-2A), zinc transporter 8 (ZnT8A; recognising arginine [ZnT8RA] or tryptophan [ZnT8WA] at position 325) and insulin (IAA, tested ≤2 weeks after diagnosis to avoid detection of antibodies to injected insulin) using up-to-date assays [9, 10]. GADA and IA-2A results were expressed as DK units/ml (assigned during the NIDDK sponsored autoantibody harmonization program [9]), whilst IAA and ZnT8A were expressed in arbitrary units (AU), derived from standard curves. Assays showed good performance in the Islet Autoantibody Standardization Program workshops (data not shown), presented at the Immunology of Diabetes Society meeting in 2012 (Victoria, BC, Canada) and 2015 (Munich, Germany).

### HLA Class II Genotyping

Mouth brush samples were genotyped for HLA class II using PCR of sequence specific oligonucleotides. HLA haplotypes of interest were designated as *DR3*-*DQ2* (*HLA*-DRB1*03-DQA1*0501 -DQB1*0201), *DR4*-*DQ8* (*HLA*-DRB1*04 -DQA1*0301 -DQB1*0302), *DR15*-*DQ6* (*HLA*-DRB1*15-DQA1*01-DQB1*0602), or none of the above (*X*). Genetic risk groups were defined as high (*DR3*-*DQ2*/*DR4*-*DQ8*), moderate (*DR3*-*DQ2*/*DR3*-*DQ2*, *DR4*-*DQ8*/*DR4*-*DQ8*, *DR4*-*DQ8*/*X*) or low risk (*DR3*-*DQ2*/*X*, *X*/*X*). Samples were also screened for the presence of *HLA*-A*24, as previously described [10].

### Data Analysis

Kendall rank tests (correlation coefficient, τ_*B*_) were used to compare UCPCR and autoantibody levels. Kruskall–Wallis and Mann–Whitney *U* tests were used to compare continuous variables within different categorical variables (e.g. genotype). χ^2^ analysis was used to compare persistence of islet autoantibodies. Analysis was carried out using the Statistical Package for the Social Sciences (SPSS), version 21 (available from www-03.ibm.com/software/products/en/spss-statistics).

## Results

### Patient Characteristics

By December 2003, 2127 individuals with longstanding diabetes had been recruited to the BOX study, of whom 1011 had suitable ‘at diagnosis’ samples available. Current contact details were available for 616 individuals and these were invited to join this study. Consent was obtained from 257 individuals, to whom sample collection kits were sent. Urine samples for UCPCR were returned by 157 participants (61.1%). Re-testing of baseline autoantibody samples was possible for 144 (47% male) of these participants of whom 132 returned follow-up serum samples (Table 1). These participants were representative of the BOX cohort for the average age at diagnosis and timing of initial sample for autoantibody testing, although there was a higher proportion of female participants.

**Table 1:** Autoantibody characteristics of participants

	Baseline	Follow-up
*n*	144	132
Male	67 (47)	59 (45)
Age at time of sample, years	11.7 (1.4–22.1)	32.9 (16.9–48.2)
Time after diagnosis, months	1.5 (−10.4–20.5)	271.8 (146.1–350.6)
Samples testing positive for islet autoantibodies:		
GADA	111 (77.1)	42 (31.8)
IA-2A	115 (79.9)	69 (52.3)
ZnT8A	104 (72.2)	14 (13.5^a^)
IAA	43 (71.7^b^)	NT
≥1 antibodies	144 (100.0)	86 (65.2)
≥2 antibodies	127 (88.2)	33 (25.0)

Data are presented as median (range) or *n* (%)

^a^For the measurement of ZnT8A at baseline, *n*=104

^b^For the measurement of IAA at baseline, *n*=60

NT, not tested

### Residual C-peptide secretion

Of the 144 urine samples tested a median of 23 years after diagnosis, 51 (35.4%) had detectable UCPCR C-peptide; seven individuals (4.9%) had residual (preserved) and 14 (9.7%) had minimal C-peptide secretion.

**Fig. 1:**
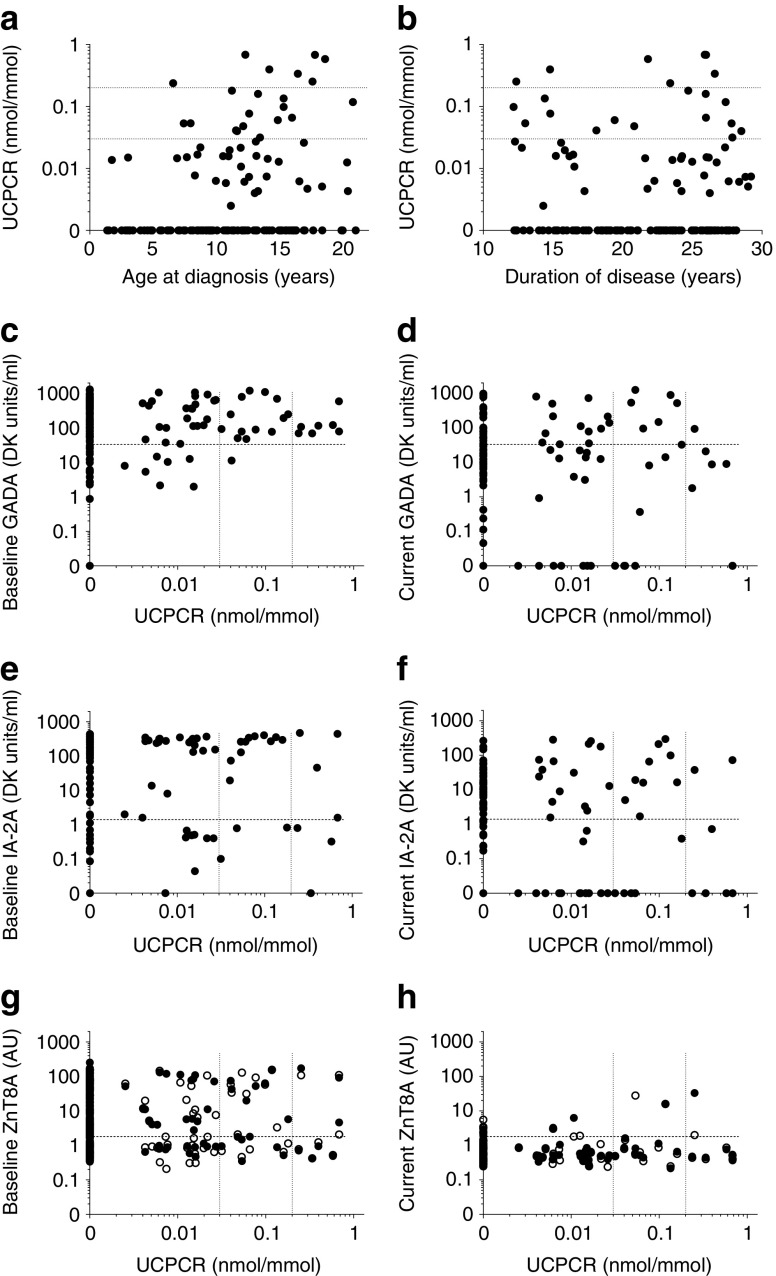
Association between UCPCR and (**a**) age at diagnosis, (**b**) duration of disease (**c**, **e**, **g**) autoantibody levels at diagnosis or (**d**, **f**, **h**) at time of UCPCR measurement. For (**g**) and (**h**) black circles, ZnT8RA; white circles, ZnT8WA. Detectable C-peptide is shown >zero nmol/mmol. Dotted lines show minimal (0.03 nmol/mmol) and residual (0.2 nmol/mmol) C-peptide levels. Dashed lines show autoantibody positivity thresholds: GADA, 33 DK units/ml; IA-2A, 1.4 DK units/ml; ZnT8A, 1.8 AU

Levels of UCPCR were associated with age at diagnosis (*p* = 0.002, τ_*B*_ = 0.198; Fig. 1a); two of 17 children (11.8%) diagnosed before the age of five had detectable C-peptide (none had minimal or residual levels) compared with 49 of 127 (38.6%) in participants diagnosed over the age of five (*p* = 0.030). Levels of UCPCR were not associated with disease duration (*p* = 0.531, Fig. 1b).

### Persistent autoimmunity in longstanding diabetes

Of the 132 participants with a follow-up serum sample, 102 (77.3%) were positive for GADA and 106 (80.3%) were positive for IA-2A at diagnosis. Of those who tested positive for GADA and IA-2A autoantibodies, 39 (38.2%) had persistent GADA and 69 (65.1%) had persistent IA-2A in the follow-up sample a median of 23 years after diagnosis. Three participants who were GADA negative at baseline became positive at follow-up. Of the 104 follow-up samples with sufficient serum for ZnT8A testing, 76 (73.1%) were positive at baseline, of which 14 (18.4%) had persistent ZnT8A.

Persistent IA-2A was more common in males than females (77% vs 56%, *p* = 0.031), while both IA-2A (median 9.1 vs 12.0 years, *p*<0.005) and ZnT8A (median 11.0 vs 15.9 years, *p* = 0.030) were associated with an older age at diagnosis.

### Antibody positivity and C-peptide

There was no association between levels of UCPCR and GADA (*p* = 0.532, Fig. 1c), IA-2A (*p* = 0.132, Fig. 1e), ZnT8RA and ZnT8WA (*p* = 0.292 and *p* = 0.894, respectively, Fig. 1g), or IAA (*p* = 0.076, data not shown) at diagnosis. Levels of UCPCR were weakly correlated with persistent GADA (*p* = 0.045, τ_*B*_ = 0.136, Fig. 1d) but not IA-2A (*p* = 0.579, Fig. 1f) or ZnT8RA and ZnT8WA (*p* = 0.660 and *p* = 0.581, respectively, Fig. 1h). Of those with residual beta cell function, five of seven were islet autoantibody negative.

### Residual beta cell function and HLA genotype

There was no association between HLA Class II risk (high, moderate or low) or *HLA*-A*24 and UCPCR level (*p* = 0.696 and *p* = 0.942, data not shown).

## Discussion

We have shown that residual endogenous insulin secretion and persistent autoimmunity occur more than a decade after diagnosis in patients with proven autoimmune-mediated type 1 diabetes. Preservation of C-peptide was associated with age at diagnosis but not with disease duration; none of those diagnosed before the age of 5 years had residual beta cell function (equivalent to stimulated serum C-peptide more than 0.2 nmol/l) compared with a fifth of those diagnosed after this age. However, no correlation was found between C-peptide and persistent autoimmunity.

This is the first study to investigate endogenous insulin secretion in longstanding diabetes using islet autoantibody data at time of diagnosis. Up-to-date assays were used to test 144 historical and current samples for UCPCR and for islet autoantibody (GADA, IA-2A, ZnT8A) status. Additionally, IAA was measured but, since most samples were taken after insulin treatment had begun, these autoantibodies were only tested in 60 participants at baseline. Ongoing islet autoimmunity was evident in >60% of participants, with particular persistence of IA-2A. The reason why some individuals remain islet autoantibody positive for decades while others do not, is partly related to age at onset of diabetes and sex.

Of seven individuals with residual beta cell function, five had no evidence of ongoing islet autoimmunity. Correlation of C-peptide levels with current GADA levels only achieved borderline statistical significance. Overall, we did not see a strong relationship between baseline autoantibody responses and persistence of beta cell function. A larger longitudinal study is required to investigate this further.

A recent study by Oram and colleagues [8], used the same UCPCR assay as that used in this study to report that 740 of 924 (80%) participants in a cross-sectional study had detectable levels of exogenous C-peptide, a median of 19 years from diagnosis. This is more than double the frequency of detectable C-peptide observed in our study. While the participants in our study had confirmed type 1 diabetes at diagnosis, this does not account for the large disparity in findings. Despite looking closely at the participant characteristics and sample collection protocols for each study, an explanation for the difference between the studies could not be identified. Data were not available from this study to examine whether detectable C peptide secretion is associated with improved glycaemic control. The finding of detectable C-peptide and persistent islet autoimmunity supports histological observations of intact beta cells in the pancreas from individuals with longstanding type 1 diabetes [1]. The presence of functioning beta cells years after diagnosis, and the strong association between age at diagnosis and endogenous insulin secretion suggest that, compared with those who have later onset of the disease, the aggressive autoimmune response associated with early onset type 1 diabetes may be more difficult to regulate, resulting in complete beta cell destruction. Intrinsic differences in immune function of those with measurable beta cell function, compared with those without is worthy of further analysis. It might be postulated that those with residual beta cell function have more effective T cell regulation.

In summary, these findings demonstrate that, in longstanding type 1 diabetes, there is evidence of ongoing autoimmunity in the majority of individuals. However, in some (particularly in individuals diagnosed above age 5), endogenous insulin secretion may continue for years after diagnosis, indicating that some beta cells may be protected from continued autoimmune attack.

## Abbreviations

AU: Arbitrary units
BOX study: Bart’s Oxford study
GADA: GAD autoantibody
IA-2A: Islet antigen-2 autoantibody
IAA: Insulin autoantibody
UCPCR: Urine C-peptide:creatinine ratio
ZnT8A: Zinc transporter 8 autoantibody
ZnT8RA: Zinc transporter 8 autoantibody recognising arginine
ZnT8WA: Zinc transporter 8 autoantibody recognising tryptophan
